# Durable clinical benefit from gefitinib plus bevacizumab in a non-small cell lung cancer patient with an acquired C797S mutation following osimertinib treatment: a case report and literature review

**DOI:** 10.3389/fonc.2025.1673836

**Published:** 2025-10-13

**Authors:** Yuping Tan, Meng Dong, Xi Li, Wenbo Li, Fei Xiong, Weidong Bao, Yongzhou Dai, Linjun Yue, Yuan Liu

**Affiliations:** Department of Interventional Oncology, Hospital of Chengdu University of Traditional Chinese Medicine, Chengdu, China

**Keywords:** non-small-cell lung cancer, epidermal growth factor receptor, C797S mutation, tyrosine kinase inhibitor, bevacizumab

## Abstract

**Background:**

The emergence of the epidermal growth factor receptor (EGFR) C797S mutation in the absence of T790M represents a common mechanism of acquired resistance to first-line osimertinib in non-small cell lung cancer (NSCLC), posing a significant clinical challenge.

**Case presentation:**

We present the case of a 72-year-old man diagnosed with metastatic NSCLC harboring an EGFR exon 19 deletion who developed an acquired C797S mutation (without T790M) following 25 months of first-line osimertinib therapy. Based on this molecular profile, the patient received a regimen of gefitinib combined with bevacizumab. This combination was well tolerated and resulted in a partial response, achieving a progression-free survival (PFS) of 15.5 months.

**Conclusion:**

This case indicates that the combination of gefitinib and bevacizumab may offer a durable clinical benefit for NSCLC patients who develop osimertinib resistance via the C797S mutation. The observed outcomes warrant further investigation into this dual-blockade strategy, especially given the limited duration of response noted for tyrosine kinase inhibitor (TKI) monotherapy.

## Introduction

Although osimertinib, a third-generation epidermal growth factor receptor (EGFR) tyrosine kinase inhibitor (TKI), is the standard first-line treatment for metastatic non-small cell lung cancer (NSCLC) with activating EGFR mutations (1–4), the development of acquired resistance inevitably limits its long-term efficacy (1). Among the on-target resistance pathways, the acquisition of the EGFR C797S mutation is one of the most common mechanisms of resistance (3, 4). This mutation results in the substitution of a critical cysteine residue in the ATP-binding site, which prevents the formation of the covalent bond essential for the irreversible activity of TKIs. Following failure of first-line osimertinib, C797S typically emerges in the absence of T790M, creating a molecular profile that restores sensitivity to first-generation reversible TKIs. Conversely, when C797S co-occurs with T790M, the allelic configuration becomes crucial: the *trans* configuration remains sensitive to a combination of first- and third-generation TKIs, while the predominant *cis* configuration confers broad resistance to all currently approved EGFR-TKIs. Current strategies to address this challenge are limited.

Treating EGFR C797S mutations without T790M with a first-generation TKI appears to be a logical approach. Clinical evidence from published case reports indicates that this strategy yields a variable duration of benefit, with progression-free survival (PFS) typically ranging from 4 to 8 months, although longer responses have occasionally been reported (5–7). The relatively brief duration of these responses suggests that even in the absence of T790M, tumor cells can develop alternative resistance mechanisms to single-agent TKI therapy. This observation underscores the necessity for more robust strategies that can proactively address these potential resistance pathways. One such strategy involves combining an EGFR-TKI with an antiangiogenic agent, such as bevacizumab. The rationale for this approach is supported by well-documented “crosstalk” between the EGFR and Vascular Endothelial Growth Factor (VEGF) signaling pathways. Activated EGFR can upregulate VEGF expression, which can subsequently function as a bypass signaling pathway to sustain tumor growth and mediate resistance to TKI therapy (8–10). The clinical benefit of this synergistic effect has been firmly established in the first-line setting, where the addition of bevacizumab to an EGFR-TKI significantly prolonged PFS in several pivotal clinical trials (11, 12). However, whether this combination can effectively overcome acquired resistance and provide durable benefits in the specific post-osimertinib, C797S-only context remains an important clinical question.

Herein, we present a case of an EGFR-mutant NSCLC patient with an acquired C797S mutation without T790M resistance to osimertinib who achieved prolonged disease control with gefitinib plus bevacizumab. To investigate the factors contributing to this durable response, we systematically reviewed and compared this outcome with those reported in the literature for similar molecular profiles treated with alternative strategies. By summarizing the potential mechanisms of action and comparing our clinical outcomes with existing evidence, this study aimed to provide a clinical reference and conceptual framework for future therapeutic strategies in the complex landscape of post-osimertinib treatment involving the C797S mutation without T790M.

## Case presentation

**Figure 1:**
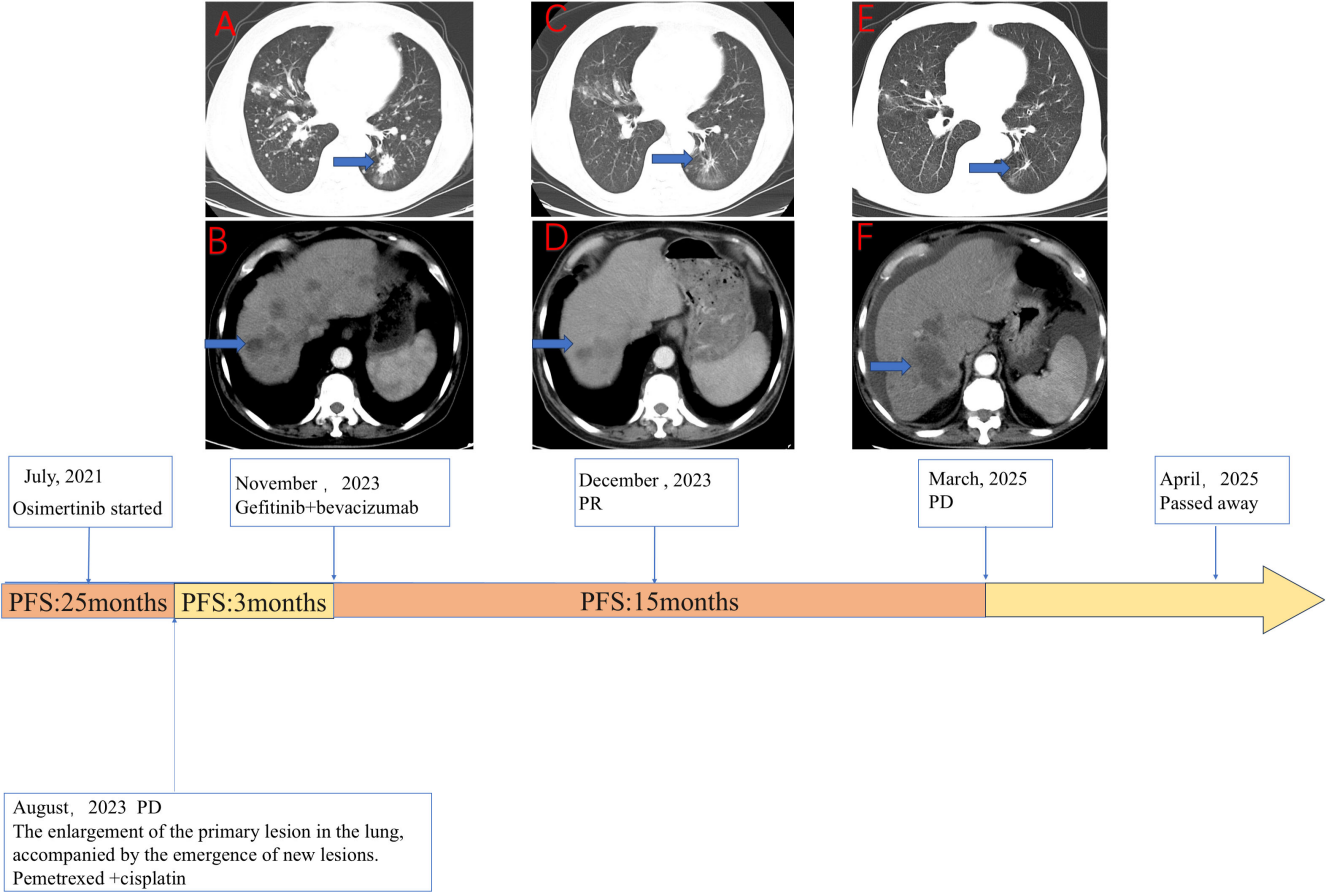
The clinical course of the patient detailing the sequence of therapies alongside the corresponding radiological evolution of the disease. **(A, B)** Axial computed tomography (CT) images of the chest **(A)** and abdomen **(B)** taken in November 2023 depict the baseline status prior to the initiation of gefitinib combined with bevacizumab. These images reveal a significant tumor burden, characterized by a primary lesion in the left lung (**A**, blue arrow) and multiple liver metastases (**B**, blue arrow). **(C, D)** Follow-up CT scans from December 2023, conducted 1 month after the commencement of gefitinib plus bevacizumab treatment, demonstrate a marked reduction in the size of both the lung lesion (**C**, blue arrow) and the liver metastasis (**D**, blue arrow), indicative of a partial response (PR). **(E, F)** CT scans from March 2025 illustrate disease progression (PD) occurring after 15.5 months of therapy, with evident regrowth of the target lesions in the lung (**E**, blue arrow) and liver (**F**, blue arrow). The timeline at the bottom provides a summary of the patient’s therapeutic journey. The patient received first-line osimertinib for 25 months before experiencing disease progression (PD) in August 2023. Following a 3-month course of bridging chemotherapy with pemetrexed and cisplatin, treatment with gefitinib plus bevacizumab commenced in November 2023. This combination regimen resulted in a progression-free survival (PFS) of 15.5 months. The patient ultimately passed away in April 2025.

In July 2021, a 72-year-old man with a significant history of smoking was diagnosed with metastatic lung adenocarcinoma (Stage T4N2M1, 8th Edition Union for International Cancer Control (UICC)), exhibiting metastases to the contralateral lung, mediastinal lymph nodes, and liver. Molecular profiling of the tumor tissue revealed an EGFR exon 19 deletion (19del) and T790M-negative NSCLC. The patient initiated first-line treatment with osimertinib, which resulted in a durable clinical benefit lasting 25 months. However, in August 2023, routine imaging confirmed disease progression. Subsequently, he underwent three cycles of chemotherapy with pemetrexed and cisplatin, which were complicated by Grade 3 myelosuppression; this condition was resolved with supportive care. In November 2023, he was admitted to our institution, presenting with respiratory symptoms. A contrast-enhanced CT scan demonstrated extensive disease, including a 2.8 cm × 1.7 cm primary mass in the left lower lobe, along with pulmonary, pleural, bone, liver, adrenal, and peritoneal metastases (**Figure 1**).

To elucidate the mechanism of resistance, we performed a percutaneous liver biopsy. Next-generation sequencing (NGS) analysis confirmed the persistence of the original EGFR 19del mutation and identified a newly acquired EGFR C797S mutation. Notably, the T790M mutation was absent, and no other known resistance bypass pathways were detected. Based on this molecular profile, a combination regimen of gefitinib (250 mg daily) and bevacizumab (15 mg/kg every 3 weeks) was initiated on November 30, 2023. The patient experienced significant symptomatic improvement, and the initial radiological assessment confirmed a partial response (PR) according to Response Evaluation Criteria In Solid Tumors (RECIST) v1.1 (**Figure 1**). The treatment was well tolerated, with the primary adverse event being Grade 2 diarrhea, which was managed symptomatically. He continued this combination therapy until disease progression was documented on March 17, 2025 (**Figure 1**), achieving a PFS of 15.5 months with this regimen. Following the emergence of new metastatic lesions in the liver, the primary tumors and metastatic lesions in the lungs remained in PR. The patient was subsequently offered symptomatic supportive treatment but declined all systemic therapies and passed away from septic shock secondary to peritonitis on April 1, 2025, with an overall survival (OS) of 44 months from the initial diagnosis.

## Discussion

The emergence of the EGFR C797S mutation in the absence of T790M following osimertinib therapy presents a significant clinical challenge in NSCLC (1, 2). This on-target alteration is one of the most common mechanisms of resistance to third-generation EGFR TKIs (3, 4). Following the progression of osimertinib, standard platinum-based chemotherapy remains the most common treatment option; however, its clinical benefits are limited. A substantial real-world study indicated a median overall survival of merely 11.4 months in this context, underscoring the considerable limitations of existing conventional therapies (13). Given the relatively severe toxic side effects and limited efficacy of chemotherapy, TKIs appear to be a more favorable option.

The molecular basis for C797S-mediated resistance to osimertinib is well-defined. Osimertinib, an irreversible third-generation TKI, functions by forming a covalent bond with the cysteine residue at position 797 within the ATP-binding site of the mutant EGFR. The substitution of this cysteine with a serine (C797S) eliminates the necessary anchor point for this covalent bond, thereby rendering osimertinib and other irreversible TKIs ineffective (14, 15). Crucially, this structural change does not impair the binding of first-generation reversible TKIs, such as gefitinib and erlotinib, which do not rely on covalent bonding. Preclinical models have consistently demonstrated that cells harboring a C797S mutation in the absence of T790M remain sensitive to first-generation TKIs while exhibiting resistance to third-generation agents (16). This biological concept underpins the strategy of treating patients with a first-generation TKI who develop this specific resistance profile. Previous studies have reported that NSCLC harboring EGFR mutations, particularly those with acquired resistance mediated by a T790M and *trans*-C797S configurations, are sensitive to a combination therapy of a first-generation EGFR-TKI plus osimertinib (17–20). While the *trans* configuration of T790M and C797S may respond to a combination of first- and third-generation TKIs, the *cis* configuration presents a significantly greater challenge. Tumors exhibiting the T790M *cis* with C797S profile are resistant to all approved EGFR-TKIs, thereby creating a substantial therapeutic gap. Consequently, most research on targeted agents for this population remains in the preclinical or early clinical development stages (21–23). With the mere presence of a C797S mutation without T790M, first-generation TKI was recommended by several studies (5–7, 16). A detailed analysis of the cases presented in **Table 1** is warranted to understand the potential factors contributing to the variability in treatment outcomes following first-generation TKI treatment. Published reports consistently demonstrate that the PFS achieved with TKI monotherapy for C797S-only mutations is modest, typically ranging from 4 to 8 months, with occasional reports of longer durations (5–7, 16). This limited efficacy ceiling cannot be solely attributed to the intrinsic activity of the drug; rather, it is more likely associated with tumor heterogeneity and the complex resistance microenvironment.

**Table 1:** Comparison of the present case with published cases of first-generation TKI therapy for C797S-positive/T790M-negative NSCLC after third-generation TKI resistance.

Study	Patient characteristics	Prior 3rd-Gen TKI	Acquired mutation	Subsequent treatment	Progression-free survival (PFS)
Present case	72-year-old man, 19del	Osimertinib	C797S	Gefitinib + bevacizumab	15.5 months
Jin X, et al. (6).	55-year-old woman, 19del	Almonertinib	C797S	Icotinib (monotherapy)	8 months
Muscolino P, et al. (7).	Case #1: 62 y/o F, L858R	Osimertinib	C797S	Gefitinib + stereotactic body radiotherapy	18+ months (stable disease in the extracranial lesions)
	Case #2: 69 y/o M, 19del	Osimertinib	Not specified¹	Gefitinib (monotherapy)	8 months
	Case #3: 60 y/o M, 19del	Osimertinib	Not specified¹	Gefitinib (monotherapy)	8+ months (ongoing)
Enrico D, et al. (16).	66-year-old man, 19del	Osimertinib	T790M loss + C797S	Gefitinib (monotherapy)	4 months (ongoing at time of report)
Cai F, et al. (5).	68-year-old woman, 19del	Osimertinib	C797S	Icotinib (monotherapy)	8 months

F, female; M, male; y/o, year-old; 19del, exon 19 deletion; L858R, p.Leu858Arg mutation; PFS, progression-free survival; TKI, tyrosine kinase inhibitor; NSCLC, non-small cell lung cancer.

**Figure 2:**
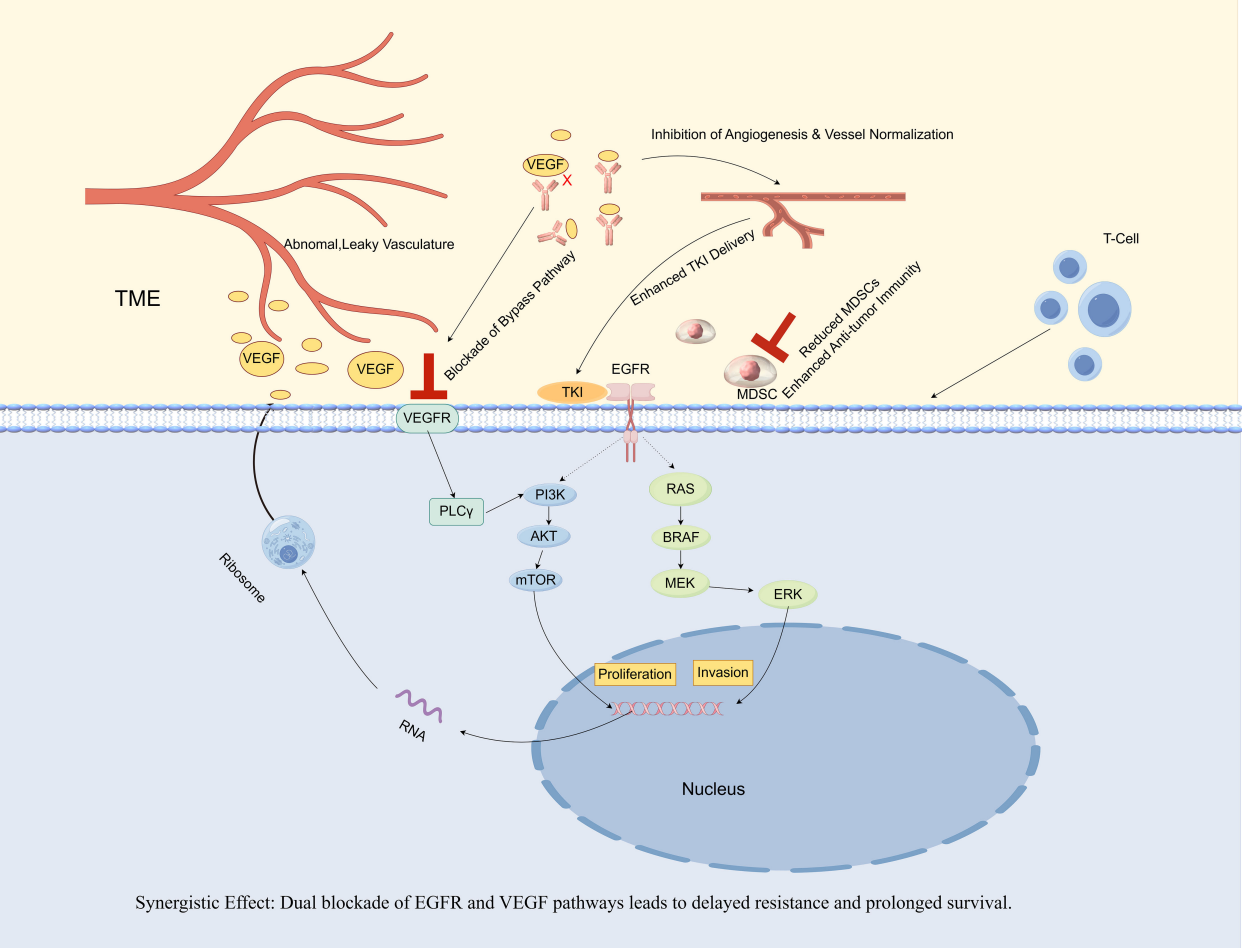
Synergistic antitumor mechanisms of gefitinib and bevacizumab combination therapy in *EGFR*-mutant non-small cell lung cancer (NSCLC).

The PFS of 15.5 months achieved with the combination of gefitinib and bevacizumab in our case indicates a significant improvement. This enhancement may be attributed to several mechanisms that are supported by existing literature (**Figure 2**).

The combination of an EGFR-TKI (gefitinib) and an anti-VEGF antibody (bevacizumab) establishes a synergistic approach. Gefitinib directly inhibits the oncogenic signaling associated with the mutant EGFR, while bevacizumab concurrently neutralizes VEGF, which is frequently upregulated by EGFR signaling (8). This dual blockade results in four significant synergistic effects. 1) Tumor vasculature is inhibited and normalized, thereby enhancing the delivery of gefitinib and oxygen to the tumor (24, 25). 2) The VEGF/Vascular Endothelial Growth Factor Receptor (VEGFR) bypass pathway is blocked, a prevalent mechanism of TKI resistance (9). 3) It remodels the Tumor Mircroenvironment (TME). Mechanistically, VEGF acts as a chemoattractant and expansion factor for myeloid-derived suppressor cells (MDSCs) by signaling through VEGFRs expressed on these cells. By neutralizing VEGF, bevacizumab disrupts this axis, thereby reducing the recruitment and accumulation of immunosuppressive MDSCs and restoring an effective anti-tumor immune response, as evidenced by an increase in effector T cells (26). 4) VEGF can initiate an autocrine loop by binding to its own receptors (VEGFRs) located on the surface of tumor cells. This mechanism directly enhances tumor cell proliferation in a manner that is independent of angiogenesis, thereby complementing the anti-apoptotic survival signals mediated by the EGFR pathway. The synergy between these pathways underscores the efficacy of a dual-blockade strategy targeting both pathways. This comprehensive inhibition of both tumor cells and their microenvironment results in superior efficacy and prolonged survival, as evidenced by clinical trials (11, 12) and supported by the findings of the present case.

The direct inhibition of VEGF-mediated bypass signaling is critical. The VEGF pathway can mediate acquired resistance to EGFR-TKIs by providing an alternative route for downstream signal activation. Specifically, VEGF/VEGFR signaling can reactivate key pro-survival pathways, such as PI3K/AKT and MAPK, even when EGFR is effectively inhibited. Moreover, the research indicates that bevacizumab effectively counteracts VEGF-dependent resistance to erlotinib (9). Preclinical studies have confirmed that when the EGFR pathway is inhibited, tumor cells can become dependent on VEGF signaling for survival and proliferation. Therefore, it is plausible that the early introduction of bevacizumab in our case directly suppressed this critical bypass pathway, thereby preventing the growth of resistant clones that could have led to early failure with monotherapy. In addition, another highly important mechanism is the sustained suppression of latent resistant clones via microenvironment modulation. Studies by Jin et al. and Rangachari et al. revealed that the re-emergence of T790M is a key mechanism of failure for monotherapy (6, 10). The tumor microenvironment, particularly hypoxia, is recognized as a potent driver of TKI resistance and can promote the selection of preexisting resistant clones, such as T790M-positive cells (17). A major mechanism of anti-angiogenic agents like bevacizumab is the normalization of tumor vasculature, which improves perfusion and alleviates tumor hypoxia (24, 25). However, a growing body of evidence indicates that the interplay between the EGFR and VEGF/VEGFR pathways extends beyond this, involving a direct autocrine signaling loop within the tumor cells themselves (27). A landmark study by Lichtenberger et al. (2010), published in *Cell*, elucidated this core mechanism using sophisticated genetically engineered mouse models. The study revealed that in tumor cells with an activated EGFR pathway, a multi-layered interplay exists. Initially, activated EGFR signaling, via the downstream Extracellular Regulated Protein Kinases (ERK) pathway, not only promotes the secretion of the VEGF ligand but also upregulates the expression of VEGF receptors on the surface of the tumor cells themselves. Subsequently, the secreted VEGF acts back on these receptors, establishing an autocrine loop that directly stimulates tumor cell proliferation independently of angiogenesis. This provides direct evidence for VEGF’s role as a potent growth factor for tumor cells. Furthermore, the study demonstrated a profound synergy between these two pathways within the neoplastic cells. The EGFR pathway primarily provides an anti-apoptotic survival signal, while the autocrine VEGF/VEGFR loop contributes a pro-proliferative signal (28). Critically, while blocking either pathway alone resulted in significant tumor growth inhibition, simultaneously blocking both pathways led to a substantially greater antitumor effect.

The durable response to gefitinib plus bevacizumab in our patient suggests a potential synergy. We hypothesize that the normalization of tumor vasculature and the subsequent alleviation of tumor hypoxia by bevacizumab may serve as a key upstream event that contributes to multiple downstream therapeutic benefits. This improved perfusion could plausibly enhance the delivery of gefitinib, thereby increasing its effective concentration at the target site. Concurrently, the reduction of hypoxia may have contributed to remodeling the immunosuppressive tumor microenvironment, potentially by decreasing the recruitment of immunosuppressive cells like MDSCs. Alleviating hypoxia may also attenuate the activation of hypoxia-inducible bypass signaling pathways, possibly delaying the development of acquired resistance.

Therefore, we propose a model where hypoxia reduction could act as a central mechanism linking the anti-angiogenic effects of bevacizumab to the observed synergistic benefits. This conceptual framework offers a plausible, albeit unproven, explanation for the prolonged PFS in our case and highlights the multifaceted role of anti-angiogenic therapy, which warrants further mechanistic investigation. The potential influence of baseline patient characteristics and inter-patient differences must be considered as potential contributors to treatment outcomes. Factors such as tumor burden, sites of metastasis, and the presence of undetected co-mutations are known to influence the efficacy of TKIs. Specifically, co-mutations in genes such as TP53 have been associated with poorer prognoses in patients receiving EGFR-TKIs (29). While our patient may have lacked these negative prognostic factors, this cannot be definitively confirmed due to the limitations of the genomic profiling conducted.

In summary, due to the case report nature of this study, some limitations need to be considered. The absence of molecular biology experimental validation was also one of the limitations of our study. A tissue biopsy was performed in a liver metastatic lesion, and molecular heterogeneity between the primary and metastatic lesions was observed. Lung adenocarcinoma displays considerable intratumoral heterogeneity, characterized by primary tumors that consist of multiple subclones, each harboring distinct genetic alterations. Key driver mutations, such as EGFR and KRAS, are generally clonal and emerge early in tumor evolution, being present in both primary and metastatic sites. Conversely, subclones with subsequent genomic alterations, particularly those responsible for chromosomal instability, are often enriched in metastatic lesions, resulting in genomic discordance between the primary tumor and its metastases (30). It is crucial to emphasize that, as a single case report, our findings are hypothesis-generating and should not be regarded as definitive. This underscores the multifaceted role of anti-angiogenic therapy, which necessitates further mechanistic investigation.

Despite these limitations, while a first-generation TKI monotherapy offers a viable therapeutic path for patients with C797S mutations, its efficacy appears to be limited by tumor heterogeneity and microenvironment-driven clonal evolution. The outcomes in our case suggest that the early introduction of bevacizumab in a combination regimen, through its multifaceted mechanisms, may be a feasible strategy to overcome this efficacy ceiling. When we compare our approach to other therapeutic options, its clinical value appears to be favorable. The PFS in our case exceeds the typical outcomes of standard chemotherapy (13). Furthermore, while fourth-generation TKIs such as BBT-176 (31) and BLU-945 (32) are under development, our regimen utilizes established agents, providing an immediately actionable option for patients in need today.

## Conclusion

In conclusion, this case report presents clinical evidence that the combination of gefitinib and bevacizumab may achieve a durable response in a patient with NSCLC who developed resistance to osimertinib via a C797S mutation. The observed outcome, particularly when compared to historical data for first-generation TKI monotherapy, supports the rationale for further exploration of this dual-blockade strategy as a potential therapeutic option for this specific clinical scenario. Looking ahead, the ongoing clinical development of fourth-generation TKIs, specifically BLU-945 and BBT-176, represents a highly promising future direction for overcoming C797S-mediated resistance and enhancing outcomes for this patient population.

## Data Availability

The raw data supporting the conclusions of this article will not be made publicly available due to privacy and ethical restrictions, as they contain information that could compromise patient confidentiality. De-identified data may be made available by the corresponding author upon reasonable request.
